# Telehealth, Telebehavioral Health, and Patient Portal Willingness, Use, and Access Challenges among Migrant Farmworkers on H-2A Visas, North Carolina, USA, 2024

**DOI:** 10.1007/s10903-025-01767-x

**Published:** 2025-08-26

**Authors:** Elisabeth C. Reed, Nowrin Nusrat, Lilibeth Andres, Tania Connaughton-Espino, Natalie D. Rivera, Modjulie A. Moore, Jose A. Robles Arvizu, Catherine E. LePrevost, Joseph G. L. Lee

**Affiliations:** 1 Department of Implementation Science, Division of Public Health Sciences, Wake Forest University School of Medicine, Winston-Salem, North Carolina United States; 2 Department of Applied Ecology, College of Agriculture and Life Sciences, North Carolina State University, Raleigh, North Carolina United States; 3 NC Farmworker Health Program, Office of Rural Health, NC Department of Health and Human Services, Raleigh, North Carolina United States; 4 Department of Family Medicine, School of Medicine, University of North Carolina at Chapel Hill, Chapel Hill, North Carolina United States; 5 Department of Health Education and Promotion, College of Health and Human Performance, East Carolina University, Greenville, North Carolina United States

**Keywords:** Telehealth, Farmworkers, Healthcare access, Digital healthcare

## Abstract

Digital health services can facilitate patients’ access to healthcare. However, access to reliable internet and utilization of digital healthcare are patterned by race, ethnicity, and class in the United States. We assessed willingness, use, and challenges accessing digital healthcare platforms among farmworkers arriving in North Carolina on temporary H-2A work visas (*n* = 327). We fielded a survey on digital healthcare access in English and Spanish at a central arrival hub for H-2A visa holders in 2024. We calculated descriptive statistics and assessed associations between willingness to use and uptake of digital healthcare and demographic variables. Most participants were interested in utilizing telehealth and patient portals, but few had ever used these services. Among participants who had used telehealth, 50% reported needing assistance. Older participants were less likely to utilize patient portals than younger participants. Future interventions should consider the unique context of migrant farmworkers in building digital health service models and provide digital skills training.

## Background

Temporary, migrant farmworkers on H-2A visas represent approximately a third of North Carolina’s (NC’s) agricultural workforce, serving a critical role in NC’s agricultural industry [1]. Farmworkers are at substantial risk of experiencing health-related problems stemming from their temporary living arrangements and the hazardous nature of agricultural work [1]. In addition to physically demanding job tasks, farmworkers face emotional stressors, such as feelings of isolation while being far from family and living in remote areas [1]. The demands of farmwork make healthcare access critical; however, accessing care can be challenging due to cost, distance to facilities, lack of transportation, long work hours, and language barriers [2].

Farmworkers often receive care from community health centers and other safety net providers [1]. Offering digital healthcare services could be an option for increasing healthcare access to farmworkers as it can reduce the need for transportation and accommodate work schedules. While digital health services have the potential to improve healthcare access among farmworkers, with limited exception [3], little research has assessed willingness or use of these services among farmworkers [2, 4]. Thus, we sought to assess willingness to use and challenges with using these services among migrant farmworkers arriving on H-2A visas in North Carolina (NC).

## Methods

### Data Collection

Trained data collectors fielded the survey in English or Spanish using Qualtrics on an iPad on April 5, April 19, and May 24, 2024, at a major H-2A arrival hub in NC. Participants provided verbal consent and received a sun protective hat upon survey completion. The East Carolina University and Medical Center IRB approved the research protocol under an exempt determination (#23-001063). To make data collection efficient and avoid order effects between telehealth use and telebehavioral health use, we employed a split questionnaire design approach [5]. Participants were randomized to questions about telehealth and patient portals or questions about telebehavioral health services.

### Measures

A full codebook of measures is in our institutional repository [6]. We assessed three demographic characteristics: age, gender, and number of seasons worked in the U.S.A. We adapted measures assessing willingness to use telehealth and telebehavioral health services from prior work [7]. We asked participants about willingness to utilize telehealth services, if they had ever participated in a telehealth visit, and, if they had, the country in which their visit occurred. We asked questions about need for assistance and problems faced while utilizing digital health services [8], including if they had assistance joining the visit or experienced technical difficulties such as internet or device problems. We asked about willingness to utilize patient portals, if they had access to patient portals, and, if applicable, from what country’s health services they had portal access. Participants who answered questions related to telebehavioral health were asked about their awareness and their willingness to use these services.

### Analysis

We generated frequency distributions for categorical variables and summary statistics for continuous variables. Outcome variables were dichotomized into either willing (willing and a little willing) or not willing. We used logistic regression models to investigate associations between age and seasons worked in the U.S.A., willingness to use digital health services, and use of services. Regarding missing data, we used pairwise deletion. Access to patient portals had missing values from 10.78% of participants; data collectors suggested this may have been due to the complexity of talking about systems and access across two countries. We used R software (v.4.4.0) to analyze data.

## Results

A total of 327 farmworkers participated in the survey. Most participants were males aged 18 to 68, with a mean age of 37.73 (SD = 10.85) (Table 1). The number of seasons worked in the U.S.A. varied from 0 to 35 (mean = 11.23, SD = 8.10).

**Table 1:** Characteristics of farmworkers participating in a survey at an H-2A visa arrival hub, North Carolina, 2024 (*n* = 327)

Demographic characteristics	*n*	%
Gender		
Male	324	99.08
Female	3	0.92
Age (in years)		
18–26	54	16.51
27–34	98	29.97
35–42	69	21.10
43–50	54	16.51
51+	52	15.90
Seasons worked prior to the current season		
None	23	7.03
1–3	39	11.93
4–6	47	14.37
7–9	53	16.21
10–12	43	13.15
13–15	29	8.87
16–20	40	12.23
21+	53	16.21

We asked 167 participants about their willingness to participate in telehealth and their use of patient portals. Of these participants, 90.42% reported willingness to participate in telehealth, but only 13.17% had ever used telehealth (Table 2). Willingness to use telehealth services was not significantly associated with age (OR 0.98, 95% CI: 0.91–1.06) or the number of seasons worked in the U.S.A. (OR 0.98, 95% CI: 0.88–1.09). Age (OR 1.06, 95% CI: 0.99–1.13) and the number of seasons worked (OR 0.93, 95% CI: 0.85–1.02) did not have a significant association with the use of telehealth services. Among participants who had used telehealth, 50% noted a need for assistance when using telehealth services. Internet connectivity issues affected a small proportion (9.09%) of users. No participants noted difficulties with their devices.

**Table 2:** Percentage of farmworkers arriving on an H-2A visa willing to participate in online or video telehealth, those who had ever used telehealth, and those willing to use a portal to access health records or test results by age and seasons worked in the U.S.A., North Carolina, 2024 (*n* = 167)

Characteristics	Telehealth willingness	Telehealth use	Portal access willingness
Age			
18–26	96.67	3.33	93.33
27–34	92.31	17.31	80.77
35–42	83.87	16.13	70.97
43–50	86.67	13.33	73.33
51+	91.67	12.50	58.33
Seasons			
None	88.89	11.11	77.78
1–3	100.00	9.09	90.91
4–6	96.67	16.67	83.33
7–9	90.32	12.90	87.10
10–12	85.00	20.00	70.00
13–15	72.73	9.09	54.55
16–20	80.00	13.33	60.00
21+	93.10	10.34	68.97

Most participants (76.65%) who were asked about patient portals reported willingness to use them. Older farmworkers were less willing to access patient portals (OR 0.94, 95% CI: 0.89–0.99). Of the 148 participants with data about the availability of patient portals, 12.84% reported accessing health records through patient portals, and 63.16% and 10.53% of patient portals were based in Mexico and in the U.S.A., respectively.

As part of our split questionnaire design, we asked 160 participants about their awareness of and willingness to use telebehavioral health services. Less than half (44.38%) of respondents had heard about telebehavioral health services and 90% expressed willingness to use these services. Willingness to use telebehavioral health services did not significantly correlate with age (OR 1.01, 95% CI: 0.93–1.10) or the number of seasons worked (OR 1.01, 95% CI: 0.90–1.12).

## Discussion

Lack of transportation, remote location, long workdays, lack of language access, and cost of care are barriers to accessing healthcare among farmworkers [2]. Digital health services could improve healthcare access, and such services could assist in monitoring ongoing conditions, delivering behavioral healthcare, and connecting farmworkers to specialists. Most participants indicated a willingness to utilize telehealth services (≥ 90%), telebehavioral health services (≥ 90%), and patient portals (> 75%). However, relatively few participants had ever used telehealth and patient portals. Less than half of participants reported hearing about telebehavioral health services in the U.S.A.

Among participants who reported use of telehealth services, half indicated needing assistance using the platform. Older participants were less likely to utilize patient portals. Older farmworkers may need additional training on how to access digital healthcare services due to limited digital skills, confidentiality concerns, and wanting to speak with a provider directly [3], which may partially explain less use among older participants.

Community health workers could help bridge this gap in digital health service use among farmworkers by providing education on how to navigate these platforms and ensuring farmworkers are aware of the availability of these resources. However, language and internet access may pose barriers to utilization, as these platforms may not always be available in languages other than English and internet access in rural regions may be inadequate. A recent study among farmworkers across NC found that 23.5% did not have consistent internet access in their residence and 51% did not have internet speeds fast enough to watch online videos [9]. Moreover, most relied on cellular devices (93.9%) and cellular data (84.9%) to access the internet [9], which may present challenges when attempting to join telehealth appointments or access patient portals.

Our findings of low utilization are concerning given the integration of health information technology into care, highlighting a longstanding finding that technology and healthcare system interventions can widen disparities given differences in resources [10]. Digital health services have the potential to bridge gaps in healthcare access for farmworker communities, but additional resources are needed to enable broad utilization of these services.

Barriers and strategies to increase utilization are worth further investigation. For example, some farmworkers could be concerned about the confidentiality of their health records, especially if they believe a breach could jeopardize opportunities to obtain future work through the H-2A program. As a potential strategy to increase utilization, artificial intelligence systems could be tested to translate platforms into a patient’s preferred language. Additionally, given the significant shortage of bilingual providers, it may be beneficial to assess virtual care platforms that allow for video-based medical interpretation services to join an encounter with the patient and provider.

### Strengths and Limitations

Study strengths include the use of a professionally translated, in-person, verbally administered survey and sampling strategy that reached migrant farmworkers at a centralized arrival hub. Limitations include the short survey length; thus, we do not have information to correlate willingness, use, and challenges with other factors, such as internet access or digital literacy skills. Additionally, this survey only included migrant farmworkers on H-2A visas, therefore omitting seasonal farmworkers and migrant farmworkers not on H-2A visas. Finally, our question about willingness may be subject to acquiescence bias, where people respond affirmatively when unsure about the answer. To reduce this, researchers recommend using a semantic differential scale (e.g., willing to not willing) [11], which we did along with using an item from prior research [7]. Nonetheless, willingness does not capture all the realities of deciding to use a technology.

## Conclusion

Participants reported high willingness to utilize digital health services, highlighting an opportunity to bring additional healthcare access to farmworker communities. However, few participants reported using these services. Future research and community efforts should focus on how internet and device access, language access, knowledge of digital health services, and digital literacy impact access and utilization of digital health services.

## Data Availability

Data are available in our institutional repository, the UNC Dataverse, doi:10.15139/S3/0PCZQR.
